# The relationship between body mass index and poor self-rated health in the South Korean population

**DOI:** 10.1371/journal.pone.0219647

**Published:** 2020-08-21

**Authors:** Eun-Seok Sung, Chang Kyun Choi, Ji-An Jeong, Min-Ho Shin

**Affiliations:** 1 Graduate School of Public Health, Chonnam National University, Gwangju, Republic of Korea; 2 Department of Preventive Medicine, Chonnam National University Medical School, Hwasun, Republic of Korea; University of North Texas Health Science Center, UNITED STATES

## Abstract

**Objective:**

This study aimed to examine the association between body mass index (BMI) and self-rated health (SRH) in Korean adults.

**Methods:**

The study included 214,997 adults who participated in the 2016 Korean Community Health Survey. Participants were categorized into four groups according to WHO Asian classification based on their BMI: underweight (<18.5 kg/m^2^), normal-weight (18.5–22.9 kg/m^2^), overweight (23.0‒24.9 kg/m^2^), obese (25.0‒29.9 kg/m^2^), and severely obese (≥30.0 kg/m^2^). Multivariate Poisson regression analysis with sampling weights and robust variance estimators was performed to evaluate the relationship between BMI categories and poor SRH.

**Results:**

A J-shaped association was observed between BMI and poor SRH in both sexes. Compared to normal-weight subjects, the age, lifestyle, and comorbidities adjusted prevalence rate ratios (PRRs) in men for poor SRH were 1.73 (95% confidence interval [CI], 1.60–1.88) for underweight, 0.87 (95% CI, 0.83–0.92) for overweight, 0.98 (95% CI, 0.93–1.03) for obese, and 1.79 (95% CI, 1.63–1.97) for severely obese. In women, compared to normal-weight subjects, the age, lifestyle, and comorbidities adjusted PRRs for poor SRH were 1.33 (95% CI, 1.26–1.41) for underweight, 1.02 (95% CI, 0.98–1.06) for overweight, 1.15 (95% CI, 1.10–1.19) for obese, and 1.42 (95% CI, 1.31–1.53) for severely obese. Associations between underweight and SRH were stronger at older ages than at younger ages, whereas those between high BMI and SRH were stronger at younger ages than at older ages.

**Conclusions:**

This cross-sectional study using a nationally representative survey observed a J-shaped relationship between BMI and poor SRH. This association differed depending on age and presence or absence of comorbidities.

## Introduction

Obesity is an important risk factor for the chronic disease burden worldwide [1–3] and is associated with increased morbidity and mortality of various chronic diseases [4]. Recently, underweight has also been associated with high all-cause and cardiovascular mortality [5–8]. However, health-related factors or diseases that mediate this association remains to be elucidated [9]. Self-rated health (SRH) is an indicator of health condition that is a composite of physical, mental, and social well-being [10]. SRH not only reflects the quality of life but also is related to a physical health condition. The association of poor SRH with high morbidity and mortality has been demonstrated not only in the general population as well as in patients with various chronic diseases [11, 12]. In addition, poor SRH is an important prognostic predictor of diseases such as cancer and heart disease [13–15].

Several studies have explored the association between body mass index (BMI) and SRH [16–21]; however, their results have been inconsistent. Some studies have reported that a higher BMI is associated with poor SRH [19, 20], whereas others have reported a U- or J-shaped association between BMI and poor SRH [16–18, 21, 22]. However, in previous studies, only the association between obesity and poor SRH was assessed, or the association between BMI and poor SRH was not properly assessed due to a lack of underweight subjects. Although there was one study in the Korean population, this one was based on a relatively small study population [23]. Many previous studies have reported sex differences in the association between BMI and poor SRH [17–19, 22]. However, few studies have evaluated the effect modification of sex in Asian populations. Therefore, the present study aimed to investigate the association between BMI and poor SRH in Korean adults and to evaluate whether this association was modified by sex.

## Material and methods

### Subjects

This study used data from the 2016 Korean Community Health Survey (KCHS), which is a detailed survey of a representative sample of the Korean population aged ≥ 19 years conducted annually since 2008 to provide health statistics at the municipality level [24]. The target population for the KCHS is adults aged ≥ 19 years living within the jurisdiction of a community health center. The stratum was divided into two stages according to the administrative unit (*Dong*, *Eup*, and *Myeon*) and housing unit (apartments and houses); the smallest administrative district unit (*Tong*, *Ban*, and *Lee*) was selected as the primary sampling unit of the stratum through probability proportionate sampling [24]. Information was collected through face-to-face interviews conducted by a trained interviewer. The 2016 KCHS included 228,452 subjects. BMI was missing for 10,371 participants (4.5%), SRH for 27 participants (<0.1%), smoking status for 6 participants (< 0.1%), alcohol consumption for 70 participants (<0.1%), physical activity for 373 participants (0.2%), marital status for 208 participants (0.1%), household income for 2,376 participants (1.0%), education level for 342 participants (0.1%), the history of hypertension for 32 participants (<0.1%), the history of diabetes for 36 participants (<0.1%), the history of stroke for 33 participants (<0.1%), the history of coronary heart disease for 126 participants (0.1%), and the history of arthritis for 76 participants (<0.1%). The final analysis included 214,997 participants (94.1%) without missing values. This study was approved by the Institutional Review Board of the Korea Centers for Disease Control and Prevention (KCDC).

### Body mass index

BMI was based on self-reported height and weight and calculated as weight in kilograms divided by height in meters squared. Correlation coefficients between the measured and self-reported BMI in the subgroup of this survey were high (r = 0.92) [25]. Participants were categorized into four groups according to WHO Asian classification [26] based on their BMI as underweight (<18.5 kg/m^2^), normal-weight (18.5–22.9 kg/m^2^), overweight (23.0–24.9 kg/m^2^), obese (25.0–29.9 kg/m^2^), and severely obese (≥30.0 kg/m^2^).

### Self-rated health

SRH was assessed based on responses to the question, “How do you rate your general health status?,” rated on a five-point scale widely used [27]. SRH was dichotomized as poor (reported as “poor” or “very poor”) or good (reported as “very good,” “good,” or “moderate”).

### Covariates

Lifestyle, socioeconomic status, and comorbidities were investigated in an interview conducted by the trained interviewee. Smoking history was coded as non-, former, or current smokers. The amount and frequency of alcohol consumption were investigated, with alcohol intake was categorized as drinker or non-drinker. Socioeconomic variables considered in this study included marital status (single, married, or divorced/bereaved/separated), educational attainment (uneducated, elementary school, middle school, high school, or college or higher), rural residency, and monthly household income (≤1.00 million, 1.01–2.00 million, 2.01–3.00 million, 3.01–4.00 million, or ≥4.01 million South Korean won). Physical activity was coded as whether subjects engaged in moderate or vigorous physical activity, regardless of whether it was performed for leisure or occupation. Moderate physical activity was defined as moderately intense physical activity (e.g., swimming at a slow pace, table tennis, badminton, tennis doubles, etc.) for more than 5 days per week for 30 min or more. Vigorous physical activity was defined as intense physical activity (e.g., swimming at a fast pace, climbing, cycling, squash, tennis singles, etc.) for more than 3 days per week for 20 min or more. Comorbidities (if respondents had doctor-diagnosed hypertension, diabetes, stroke, coronary heart disease, or arthritis) were also considered.

### Statistical analysis

**Fig 1:**
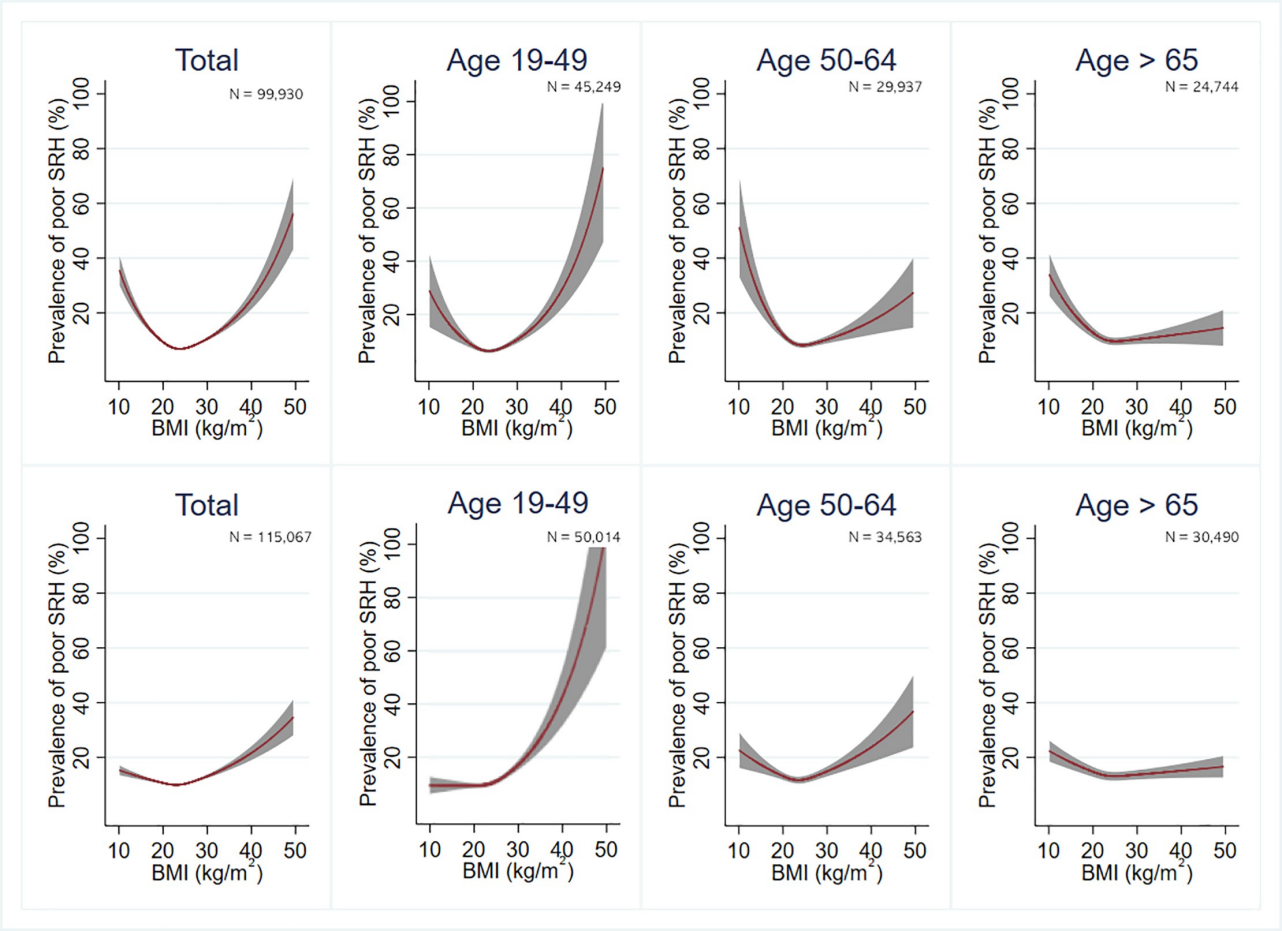
Prevalence of poor self-rated health estimated using a multivariate Poisson regression model with a restricted cubic spline according to age in men (upper row) and women (lower row). Lifestyle, socioeconomic status, and comorbidities were adjusted. The gray band represents the 95% confidence interval.

**Fig 2:**
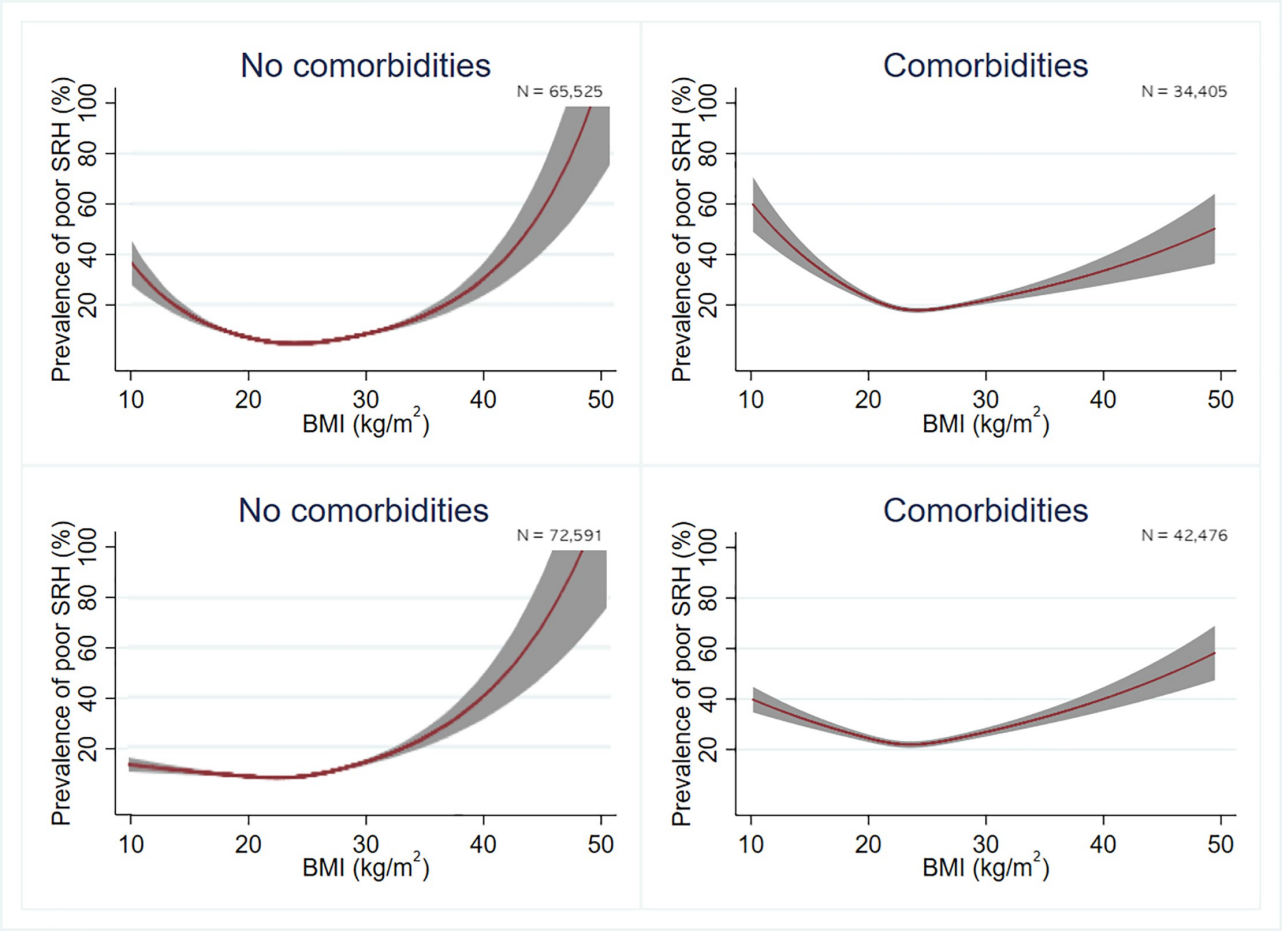
Prevalence of poor self-rated health estimated using a multivariate Poisson regression model with a restricted cubic spline according to the presence of comorbidities in men (upper row) and women (lower row). Lifestyle and socioeconomic status were adjusted. The gray band represents the 95% confidence interval.

Since there was a significant difference in the association between BMI and poor SRH between men and women, all analyses were performed separately for each sex. The relationship between BMI and SRH was assessed using multivariate Poisson regression analysis with robust variance estimators and sampling weights derived from KCHS. Because the prevalence odds ratio tends to overestimate the strength of associations [28], the association between BMI and poor SRH was assessed using the prevalence rate ratio (PRR). The normal-weight group was used as a reference. In the first model, age was adjusted. In the second model, socioeconomic status variables (household income, marital status, education level, and residence type) were added. In the third model, lifestyle variables (smoking, alcohol consumption, and physical activity) were added. In the fourth model, comorbidities (hypertension, diabetes mellitus, stroke, coronary heart disease, and arthritis) were added. Model 4 results were considered the main results. Subgroup analyses were performed according to age and comorbidities. Restricted cubic splines were used to model the nonlinear relationship between BMI and poor SRH in Figs 1 and 2 using knots at the 5th, 50th, and 95th BMI percentiles based on Harrell’s recommended percentiles [29]. Stata version 14.0 (Stata Corp, College Station, TX, USA) was used for the statistical analyses. Statistical significance was defined as *P* < 0.05.

## Results

The study population included 99,930 men and 115,067 women. Their baseline characteristics according to BMI categories are presented in Tables 1 and 2. The prevalence of underweight participants was 2.7% in men and 7.0% in women, whereas that of obesity was 3.6% and 2.5%, respectively. In men, compared to normal-weight participants, participants with underweight were older, lived alone, had lower income and education levels, lived in a rural area, engaged in less physical activity, and had lower alcohol consumption. In addition, they had a lower prevalence of hypertension but higher rates of stroke, coronary heart disease, and arthritis. However, these tendencies were reversed in participants with obesity. Compared to normal-weight men participants, participants with obesity were younger, were married, had higher income and education levels, lived in a rural area, engaged in more physical activity, and had higher alcohol consumption. In addition, they had a higher prevalence of hypertension and diabetes but lower rates of stroke, coronary heart disease, and arthritis.

**Table 1:** Baseline characteristics according to the body mass index (kg/m^2^) categories in men.

Characteristics	Underweight (<18.5)	Normal-weight (18.5–22.9)	Overweight (23.0–24.9)	Obese (25–29.9)	Severely obese (≥30)
N	2,659 (2.7)	36,115 (36.1)	27,958 (28.0)	29,629 (29.6)	3,569 (3.6)
Age (years)	59.8 ± 21.9	52.6 ± 18.0	52.6 ± 15.9	49.7 ± 15.0	41.5 ± 14.7
Marriage					
Single	636 (23.9)	8,114 (22.5)	4,501 (16.1)	5029 (17.0)	1,258 (35.2)
Married	1,685 (63.4)	24,898 (68.9)	21,295 (76.2)	22,617 (76.3)	2,130 (59.7)
Divorce/bereavement/separation	338 (12.7)	3,103 (8.6)	2,162 (7.7)	1,983 (6.7)	181 (5.1)
Household income (million won)					
≤1.00	1,049 (39.5)	7,082 (19.6)	4,199 (15.0)	3,451 (11.6)	356 (10.0)
1.01–2.00	534 (20.1)	6,462 (17.9)	4,609 (16.5)	4,362 (14.7)	495 (13.9)
2.01–3.00	401 (15.1)	6,913 (19.1)	5,453 (19.5)	6,002 (20.3)	805 (22.6)
3.01–4.00	294 (11.1)	5,902 (16.3)	4,925 (17.6)	5,584 (18.8)	723 (20.3)
≥4.01	381 (14.3)	9,756 (27.0)	8,772 (31.4)	10,230 (34.5)	1,190 (33.3)
Education					
Uneducated	192 (7.2)	875 (2.4)	413 (1.5)	317 (1.1)	24 (0.7)
Elementary	719 (27.0)	5,360 (14.8)	3,505 (12.5)	2,783 (9.4)	221 (6.2)
Middle school	392 (14.7)	4,427 (12.3)	3,392 (12.1)	3,060 (10.3)	234 (6.6)
High school	699 (26.3)	11,153 (30.9)	8,823 (31.6)	9,676 (32.7)	1,073 (30.1)
More than college	657 (24.7)	14,300 (39.6)	11,825 (42.3)	13,793 (46.6)	2,017 (56.5)
Rural residence	1,449 (54.5)	16,098 (44.6)	11,802 (42.2)	12,169 (41.1)	1,417 (39.7)
Physical activity	457 (17.2)	9,624 (26.6)	7,861 (28.1)	8,200 (27.7)	1,017 (28.5)
Smoking					
Nonsmoker	656 (24.7)	9,095 (25.2)	6,977 (25.0)	7,150 (24.1)	1,039 (29.1)
Former smoker	966 (36.3)	12,640 (35.0)	11,209 (40.1)	11,811 (39.9)	1,049 (29.4)
Current smoker	1,037 (39.0)	14,380 (39.8)	9,772 (35.0)	10,668 (36.0)	1,481 (41.5)
Alcohol intake	1,226 (46.1)	22,519 (62.4)	18,520 (66.2)	20,333 (68.6)	2,424 (67.9)
Hypertension	498 (18.7)	7,139 (19.8)	7,407 (26.5)	9,124 (30.8)	1,210 (33.9)
Diabetes	254 (9.6)	3,465 (9.6)	3,371 (12.1)	3,700 (12.5)	484 (13.6)
Stroke	101 (3.8)	955 (2.6)	699 (2.5)	638 (2.2)	56 (1.6)
Coronary heart disease	131 (4.9)	1,231 (3.4)	1,066 (3.8)	1,137 (3.8)	113 (3.2)
Arthritis	237 (8.9)	2,100 (5.8)	1,680 (6.0)	1,653 (5.6)	159 (4.5)

All values are given as n (%) or mean ± standard deviation.

**Table 2:** Baseline characteristics according to the body mass index (kg/m^2^) categories in women.

Characteristics	Underweight (<18.5)	Normal-weight (18.5–22.9)	Overweight (23.0–24.9)	Obese (25–29.9)	Severe obese (≥30)
N	7,997 (6.9)	56,964 (49.5)	24,965 (21.7)	22,225 (19.3)	2,916 (2.5)
Age (years)	47.6 ± 21.8)	50.2 ± 17.7)	55.5 ± 15.1)	56.2 ± 14.8)	52.3 ± 16.5)
Marriage					
Single	2,403 (30.0)	9,247 (16.2)	1,741 (7.0)	1,430 (6.4)	392 (13.4)
Married	3,818 (47.7)	36,548 (64.2)	17,573 (70.4)	15,403 (69.3)	1,842 (63.2)
Divorce/bereavement/separation	1,776 (22.2)	11,169 (19.6)	5,651 (22.6)	5,392 (24.3)	682 (23.4)
Household income (million won)					
≤1.00	1,863 (23.3)	10,505 (18.4)	5,560 (22.3)	5,311 (23.9)	715 (24.5)
1.01–2.00	1,034 (12.9)	8,585 (15.1)	4,526 (18.1)	4,314 (19.4)	561 (19.2)
2.01–3.00	1,257 (15.7)	9,853 (17.3)	4,471 (17.9)	4,153 (18.7)	579 (19.9)
3.01–4.00	1,194 (14.9)	9,341 (16.4)	3,991 (16.0)	3,355 (15.1)	465 (15.9)
≥4.01	2,649 (33.1)	18,680 (32.8)	6,417 (25.7)	5,092 (22.9)	596 (20.4)
Education					
Uneducated	1,025 (12.8)	4,391 (7.7)	1,881 (7.5)	1,718 (7.7)	223 (7.6)
Elementary	1,194 (14.9)	9,079 (15.9)	5,966 (23.9)	6,190 (27.9)	730 (25.0)
Middle school	367 (4.6)	5,073 (8.9)	3,594 (14.4)	3,596 (16.2)	414 (14.2)
High school	1,497 (18.7)	15,479 (27.2)	7,378 (29.6)	6,262 (28.2)	904 (31.0)
More than college	3,914 (48.9)	22,942 (40.3)	6,146 (24.6)	4,459 (20.1)	645 (22.1)
Rural residence	3,131 (39.2)	22,113 (38.8)	10,966 (43.9)	10,246 (46.1)	1,280 (43.9)
Physical activity	1,159 (14.5)	10,973 (19.3)	5,044 (20.2)	4,309 (19.4)	490 (16.8)
Smoking					
Nonsmoker	7,331 (91.7)	53,846 (94.5)	23,839 (95.5)	20,962 (94.3)	2,638 (90.5)
Former smoker	258 (3.2)	1,364 (2.4)	567 (2.3)	632 (2.8)	135 (4.6)
Current smoker	408 (5.1)	1,754 (3.1)	559 (2.2)	631 (2.8)	143 (4.9)
Alcohol intake	2,534 (31.7)	18,454 (32.4)	6,848 (27.4)	5,670 (25.5)	788 (27.0)
Hypertension	1,169 (14.6)	10,189 (17.9)	7,217 (28.9)	8,611 (38.7)	1,338 (45.9)
Diabetes	394 (4.9)	3,737 (6.6)	2,650 (10.6)	3,211 (14.4)	594 (20.4)
Stroke	125 (1.6)	814 (1.4)	437 (1.8)	543 (2.4)	81 (2.8)
Coronary heart disease	179 (2.2)	1,194 (2.1)	748 (3.0)	898 (4.0)	142 (4.9)
Arthritis	1,081 (13.5)	7,960 (14.0)	5,218 (20.9)	5,909 (26.6)	830 (28.5)

All values are given as n (%) or mean ± standard deviation.

Among female participants, compared to normal-weight participants, those with underweight were younger, more likely to be married, had lower income and education levels, lived in rural areas, were engaged in less physical activity, and had lower alcohol consumption. In addition, they had a lower prevalence of hypertension, diabetes, and arthritis. Compared to normal-weight participants, those with obesity were older, had higher income and education levels, lived in cities, and had lower alcohol consumption. In addition, they had a higher prevalence of hypertension, diabetes, stroke, coronary heart diseases, and arthritis.

Table 3 presents the PRRs for poor SRH according to BMI categories. A nonlinear relationship was observed between BMI and poor SRH. Compared to normal-weight subjects, the age-adjusted PRRs for poor SRH across BMI levels (<18.5, 23.0–24.9, 25.0–29.9, and ≥30 kg/m^2^) were 1.86 (95% CI, 1.72–2.01), 0.87 (95% CI, 0.83–0.92), 1.02 (95% CI, 0.97–1.08), and 2.33 (95% CI, 2.11–2.56), respectively, for men. Compared to normal-weight subjects, the age-adjusted PRRs for poor SRH across BMI levels (<18.5, 23.0–24.9, 25.0–29.9, and ≥30 kg/m^2^) were 1.37 (95% CI, 1.29–1.46), 1.09 (95% CI, 1.05–1.13), 1.38 (95% CI, 1.33–1.43), and 2.10 (95% CI, 1.94–2.27), respectively, for women. After further adjustment for demographic, socioeconomic, and lifestyle characteristics in model 3, this U-shaped association was slightly attenuated but remained statistically significant in both sexes. In model 4 with further adjustment for comorbidities, the PRRs of obese and severe obese were attenuated, whereas those for underweight were strengthened. Compared to normal-weight subjects, the PRRs for underweight, overweight, obsess, and severely obese were 1.73 (95% CI, 1.60–1.88), 0.87 (95% CI, 0.83–0.92), 0.98 (95% CI, 0.93–1.03), and 1.79 (95% CI, 1.63–1.97), respectively, in men, and in women, the PRR for underweight was 1.33 (95% CI, 1.26–1.41), the PRR for overweight was 1.02 (95% CI, 0.98–1.06), the PRR for obese was 1.15 (95% CI, 1.10–1.19), and the PRR for severely obese was 1.42 (95% CI, 1.31–1.53). The interaction by sex was significant (*P* for interaction < 0.001).

**Table 3:** Associations between the body mass index (kg/m^2^) categories and self-rated health.

	BMI categories	Model 1	Model 2	Model 3	Model 4
Men	Underweight (<18.5)	1.86 (1.72–2.01)	1.65 (1.53–1.79)	1.57 (1.46–1.70)	1.73 (1.60–1.88)
	Normal-weight (18.5–22.9)	1.00 (Reference)	1.00 (Reference)	1.00 (Reference)	1.00 (Reference)
	Overweight (23.0–24.9)	0.87 (0.83–0.92)	0.92 (0.88–0.97)	0.94 (0.89–0.99)	0.87 (0.83–0.92)
	Obese (25.0–29.9)	1.02 (0.97–1.08)	1.11 (1.05–1.17)	1.13 (1.07–1.19)	0.98 (0.93–1.03)
	Severe obese (≥30)	2.33 (2.11–2.56)	2.29 (2.08–2.52)	2.29 (2.07–2.52)	1.79 (1.63–1.97)
Women	Underweight (<18.5)	1.37 (1.29–1.46)	1.30 (1.23–1.38)	1.27 (1.20–1.35)	1.33 (1.26–1.41)
	Normal-weight (18.5–22.9)	1.00 (Reference)	1.00 (Reference)	1.00 (Reference)	1.00 (Reference)
	Overweight (23.0–24.9)	1.09 (1.05–1.13)	1.06 (1.02–1.11)	1.07 (1.03–1.11)	1.02 (0.98–1.06)
	Obese (25.0–29.9)	1.38 (1.33–1.43)	1.30 (1.25–1.35)	1.29 (1.25–1.34)	1.15 (1.10–1.19)
	Severe obese (≥30)	2.10 (1.94–2.27)	1.85 (1.72–2.00)	1.82 (1.68–1.96)	1.42 (1.31–1.53)

Data are presented given as ‘prevalence rate ratio (95% confidence interval)’.

Model 1 was adjusted for age.

Model 2 was additionally adjusted for socioeconomic status (household income, marital status, education level and residential area).

Model 3 was additionally adjusted for lifestyles (smoking, drinking and exercise).

Model 4 was additionally adjusted for comorbidities (hypertension, diabetes mellitus, stroke, coronary heart disease, and arthritis).

Table 4 presents the PRRs for poor SRH according to the BMI categories, stratified by age and comorbidities. In both sexes, the magnitude of the association between obesity and poor SRH decreased with increasing age. The magnitude of the association between underweight and poor SRH was smaller in the age group 65 years or older than in the young age groups. In addition, a U-shaped association between BMI and poor SRH was prominent in subjects without comorbidities compared to subjects with comorbidities.

**Table 4:** Stratified analysis of the association between the body mass index (kg/m^2^) categories and self–rated health.

		Underweight (<18.5)	Normal (18.5–22.9)	Overweight (23.0–24.9)	Obese (25.0–29.9)	Severely obese (≥30)	P for interaction
Men	Age*, years						
	19–49	1.97 (1.52–2.57)	1 (reference)	0.85 (0.75–0.98)	1.22 (1.08–1.36)	2.23 (1.92–2.60)	<0.001
	50–64	2.22 (1.85–2.67)	1 (reference)	0.89 (0.82–0.98)	0.93 (0.85–1.01)	1.20 (1.00–1.45)	
	≥65	1.49 (1.38–1.62)	1 (reference)	0.86 (0.81–0.92)	0.84 (0.79–0.90)	1.00 (0.82–1.21)	
	Comorbidity**						
	No	2.08 (1.84–2.35)	1 (reference)	0.85 (0.77–0.94)	1.08 (0.98–1.19)	2.53 (2.15–2.96)	<0.001
	Yes	1.40 (1.28–1.53)	1 (reference)	0.87 (0.82–0.93)	0.90 (0.85–0.95)	1.29 (1.15–1.45)	
Women	Age*, years						
	19–49	1.45 (1.27–1.67)	1 (reference)	1.33 (1.19–1.49)	1.68 (1.51–1.87)	2.63 (2.20–3.13)	<0.001
	50–64	1.62 (1.40–1.87)	1 (reference)	0.96 (0.89–1.03)	1.12 (1.04–1.21)	1.29 (1.13–1.48)	
	≥65	1.20 (1.13–1.27)	1 (reference)	0.93 (0.89–0.97)	0.95 (0.91–0.99)	0.94 (0.86–1.03)	
	Comorbidity**						
	No	1.50 (1.35–1.66)	1 (reference)	1.12 (1.03–1.21)	1.44 (1.32–1.57)	2.52 (2.10–3.02)	<0.001
	Yes	1.21 (1.14–1.28)	1 (reference)	0.93 (0.89–0.97)	1.01 (0.97–1.05)	1.23 (1.15–1.33)	

Data are presented as ‘prevalence rate ratio (95% confidence interval)’.

*adjusted for age, household income, marital status, education level, residence type, smoking, drinking, physical activity, hypertension, diabetes mellitus, stroke, coronary heart disease, and arthritis.

**adjusted for age, household income, marital status, education level, residence type, smoking, drinking and physical activity.

Figs 1 and 2 show the adjusted prevalence of poor SRH from restricted cubic spline models in both sexes. In both sexes, there was a J-shaped association between BMI and poor SRH, with the lowest risk observed for a BMI of 23–24.9 kg/m^2^ in both men and women. This association was more prominent in men than in women. When participants are older or accompanied by comorbidities, the association between BMI and SRH is less pronounced than those who are not.

## Discussion

This cross-sectional study using a nationwide sample demonstrated a J-shaped association between BMI and poor SRH in Korean adults. The association between BMI and poor SRH were different according to age and comorbidities.

We found that both underweight and obese were associated with poor SRH. Except for those that did not evaluate the relationship with underweight [19, 20], previous studies have reported U- or J-shaped relationships but have reported inconsistent results regarding sex-specific relationships [17, 18, 21, 22]. Heo et al. reported a J-shaped relationship between BMI and health-related quality of life [21]. Similar to the present study, when adjusted for comorbidities, the association between underweight and poor SRH was strengthened, while that between overweight and obesity with poor SRH was attenuated. In the Canadian Community Health Survey [17], BMI showed a U-shaped association with poor SRH in both sexes. In a study based on the National Health Interview Survey from 1997 to 2005 [18] and a study based on the World Health Survey (2002–2004) [22], the association between underweight and poor SRH was more evident in men whereas that between obesity and poor SRH was more evident in women. However, the Sault Antenatal Care Programme in Sweden [16] observed a U-shaped relationship in women but a linear relationship in men. This study was conducted in expectant parents, rather than in the general population, and was a small study with fewer than 1,000 subjects, which may have resulted in the discordance from the results of previous studies. Only one previous study was based on Korean. Noh et al. assessed the relationship between BMI categorized as WHO Asian classification and SRH scores, and SRH scores were the highest in the normal-weight (18.5–22.9 kg/m^2^) [23]. Compared to normal-weight, the odds ratio (OR) of poor SRH score was 1.60 in underweight, 1.08 in overweight, 1.72 in obese, and 3.97 in severely obese.

The underlying mechanism for the relationship between underweight and poor SRH can be explained as follows: first, underweight is associated with sarcopenia [30]. Subjects with sarcopenia or decreased muscle mass have lower exercise capacity and lower levels of physical activity, both of which are associated with poor SRH; second, the relationship between underweight and poor SRH could be explained by lifestyle factors and pre-existing comorbidities [31]. Although we adjusted for lifestyle factors and several comorbidities, unmeasured confounding and residual confounding is still possible to explain the association. The relationship between obesity and poor SRH may be mediated by obesity-related comorbidities. Numerous studies have reported causal relationships between obesity and cardiovascular diseases and cancer [32–34], which can lead to poor SRH and poor quality of life [35]. In our study, after adjusting for comorbidities, the strength of the relationship decreased by more than 30%. Additionally, obesity is associated with reduced physical activity and lower exercise capacity, and both conditions are associated with poor SRH.

The sex difference in the association between BMI and SRH may be partly due to differences in body fat distribution. At the same BMI, women tend to have higher body fat than men across all ages [36, 37]. In contrast, visceral adipose tissue increases more in men as BMI increases in those age < 40 years, whereas the association between visceral adipose tissue and BMI does not differ according to sex in those aged ≥ 40 years [37]. Unfortunately, as this study did not measure total body fat or lean body mass, whether the relationship between BMI and SRH varied depending on differences in fat distribution could not be assessed.

In the present study, the association between BMI and poor SRH varied with age. The associations between underweight and poor SRH strengthened with age, whereas those between the high BMI and poor SRH attenuated with age. Hulman et al. reported that the BMI trajectory of those who reported poor SRH had declined more than that of those not reporting poor SRH in subjects aged over 80 years [38]. Similar to this study, a large population-based cohort study of 3.6 million adults in the UK reported that the associations between BMI and mortality varied by age and those between high BMI and mortality attenuated with age [31].

In this study, the relationship between underweight and poor SRH was more apparent in subjects with comorbidity. These results indicate the possibility of reverse causality due to comorbidity. Our findings are consistent with previous studies. In the Scottish Health Survey, there was an inverted U-shaped association between BMI and health-related quality of life, and participants with metabolic comorbidities had poor quality of life compared to those without metabolic comorbidities across all BMI categories [39]. In prospective studies of mortality, the association between BMI and mortality was also U-shaped, and the strength of the association between underweight and mortality was greater in healthy never-smoker compared to never-smoker [40, 41].

The strength of this study is the large, nationally representative sample size. Also, since this study had a relatively high percentage (5%) of underweight, when compared to previous studies (<2.5%) [17, 18, 21], we could clarify the relationship between underweight and SRH. However, this study has some limitations. First, the cross-sectional design leads to difficulty in assessing causal relationships. Second, poor SRH could be underreported in face-to-face interviews, which could induce differential misclassification of poor SRH in participants with high or low BMI. Third, some comorbidities that could affect both self-rated health and underweight were not considered. Health conditions, such as chronic obstructive pulmonary diseases (COPD) and sarcopenia, are associated with underweight and these are also associated with quality of life.

## Conclusion

Both obesity and underweight were associated with an increased propensity for poor SRH, even after adjusting for demographic, socioeconomic, lifestyle variables and comorbidities. A J-shaped was observed between BMI and poor SRH in a nationwide sample of Korean adults. In addition, age and comorbidities modified the association between BMI and poor SRH. These results suggest that both underweight and overweight are risk factors for poor SRH. However, prospective studies using fat distribution are needed to clarify the causal relationship between these two variables.
